# Fetal alcohol spectrum disorder identification in Australia: A qualitative analysis of perspectives from psychologists and individuals with lived and living experience

**DOI:** 10.1111/acer.70040

**Published:** 2025-03-31

**Authors:** Katherine L. Kerimofski, Kirsten R. Panton, Grace Kuen Yee Tan, Carmela F. Pestell

**Affiliations:** ^1^ School of Psychological Science University of Western Australia Crawley Western Australia Australia

**Keywords:** assessment, FASD, lived experience, ND‐PAE, PAE, universal screening

## Abstract

**Background:**

Fetal alcohol spectrum disorder (FASD) is a neurodevelopmental disorder associated with prenatal alcohol exposure (PAE). In Australia, there are several barriers to assessment, including a limited number of FASD‐informed clinicians. This study aimed to understand the perspectives of psychologists, parents, caregivers, and adults with FASD on the current assessment process, as well as methods to improve FASD training and universal screening of PAE.

**Methods:**

Two groups of (1) psychologists and (2) parents, caregivers, and adults with FASD were interviewed about their experiences of FASD assessment and their recommendations for training and universal screening of PAE. Thematic analysis was employed to code data.

**Results:**

Five key themes were identified: (1) stigma and stereotypes of PAE, (2) support for universal screening of PAE, (3) differential, co‐occurring, and missed diagnoses, (4) lack of support following diagnosis, and (5) need for improved training for psychologists. Stereotypes of women who drink were present across themes, with both groups discussing the importance of PAE assessment for all women during antenatal care and when presenting for assessment of neurodevelopmental disorders. The importance of training more FASD‐informed clinicians who can understand the uniqueness of each individual with FASD was highlighted, with hopes of improving diagnostic capacity as well as support offered by psychologists.

**Conclusions:**

Recognition of the impact of PAE is growing in Australia; however, there is a need to embed this topic within university training for psychologists.

## Graphical abstract

Psychologists and parents, caregivers and adults with fetal alcohol spectrum disorder (FASD) were interviewed about their experiences of FASD assessment, their recommendations for training and universal screening of prenatal alcohol exposure (PAE). Five themes were identified: (1) stigma and stereotypes of PAE, (2) support for universal screening of PAE, (3) differential, co‐occurring, and missed diagnoses, (4) lack of support following diagnosis, and (5) need for improved training. This study highlighted the importance of training more FASD‐informed clinicians who can understand the uniqueness of each individual with FASD.

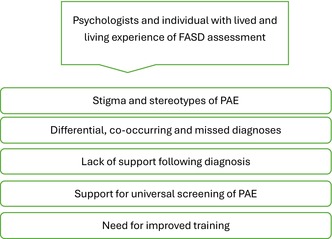

## INTRODUCTION

### Experiences and perspectives of fetal alcohol spectrum disorder assessment

Prenatal alcohol exposure (PAE) is an environmental risk factor for the development of fetal alcohol spectrum disorder (FASD; Bower et al., 2016). Alcohol acts as a teratogenic agent and can have adverse and varying impacts on a developing fetus depending on the amount, frequency, and timing of consumption, as well as maternal factors, such as age, genetics, body composition, and metabolism of alcohol (Aiton, 2021; Bower et al., 2017). There is no safe limit of PAE established in the literature, and the risk of harm to the fetus is higher with increased frequency and amount of PAE (National Health and Medical Research Council, 2020).

In Australia, FASD is a recognized disability, with the Australian Guidelines for Assessment and Diagnosis of FASD (Bower et al., 2016) currently under review following initial stakeholder consultation (Hayes et al., 2022) and public consultation in April 2024 (FASD Hub Australia, 2024). The estimated global prevalence of FASD is 7.7 cases per 1000 individuals in the general population (Lange et al., 2017; Popova et al., 2017, 2018). In Australia, higher prevalence rates have been established in a juvenile justice setting (36%; Bower et al., 2018) and in one remote Aboriginal community (12%; Fitzpatrick et al., 2015), with some calls for focused screenings for FASD in these populations, as well as children in out‐of‐home care (Fogliani, 2019; Popova et al., 2023). It is important to note FASD can occur in families from all types of cultural backgrounds and psychosocial contexts (Bower et al., 2017; Connor et al., 2020).

It has recently been established that Australian psychologists are broadly familiar with FASD as a diagnostic term and are aware there is no safe time or amount of alcohol to consume in pregnancy (Kerimofski et al., 2024). However, the majority of these psychologists were not confident in their ability to assess PAE, and despite feeling more confident in their ability to apply the diagnostic criteria for other neurodevelopmental disorders compared with FASD, they were largely unaware of clinical guidelines, as well as state and federal government recommendations to screen for PAE in assessments for intellectual disability (ID), learning difficulties, and behavioral concerns (FASD Model of Care, Department of Health, State of WA, 2010; National FASD Strategic Action Plan 2018–2028, Department of Health, 2018; Kerimofski et al., 2024). Despite the well‐established link between PAE and disruption to neurodevelopment (DeJong et al., 2019), Australian psychologists surveyed reported not wanting to shame the biological mother and not knowing how to assess for PAE as the main reasons for not asking about PAE during clinical interviews (Kerimofski et al., 2024).

Stigma and stereotypes have developed about biological mothers and individuals with FASD (Roozen et al., 2020), including public stigma about biological mothers being seen as morally culpable (Roozen et al., 2020) and some condemned as “child abusers” who “lack empathy” (Corrigan et al., 2019, p. 172). This stigma is also seen in the false stereotype that FASD is solely an “Indigenous problem” (Jandu Yani U Project Team, 2019, p. 8). The National Drug Strategy Household Survey [NDSHS] 2022–2023 (2024) highlighted that alcohol consumption in pregnancy is declining in Australia, from 42% of women aged 14–49 in 2013 to 28% in 2022–2023. Of those who consumed alcohol in pregnancy, most did not know they were pregnant, but 15% continued to consume alcohol once aware of their pregnancy (NSDHSD, 2024). Women who continued to consume alcohol in pregnancy tended to be older, have higher incomes, education, and socioeconomic status (Anderson et al., 2013; Hutchinson et al., 2013; Kingsbury et al., 2015; McCormack et al., 2017).

There are a number of barriers to FASD assessment and diagnosis, including the aforementioned public stigma (Bell et al., 2016; Howlett et al., 2019; Mukherjee et al., 2015), as well as a lack of sufficient training in FASD (McCormack et al., 2022), limited diagnostic capacity (Commonwealth of Australia, 2021) and limited referral pathways (Panton et al., 2022). A further difficulty in diagnosing FASD is the overlap with other neurodevelopmental conditions (Tanfield et al., 2024). Attention deficit hyperactivity disorder (ADHD) is the most common comorbid neurodevelopmental disorder (52.9%; Lange et al., 2018), followed by ID (23%; Weyrauch et al., 2017). Autism was found to be comorbid with FASD at a 2.9% rate (Lange et al., 2018), although this may be an underrepresentation as a recent meta‐analysis indicated this figure could be closer to 7% when looking at individuals who have PAE but no FASD diagnosis (Clark et al., 2024). Chasnoff et al. (2015) studied children who were fostered and adopted and found that 80% of children had a missed diagnosis of FASD, with their PAE unrecognized prior to the study's multidisciplinary assessments.

Individuals with FASD, as well as their parents and caregivers, have expertise in their lives and offer valuable insights into the assessment process and ways to improve service delivery. Hayes et al. (2023) conducted a systematic review of studies based on lived experiences of FASD diagnostic assessments with four overarching topics: (1) preassessment concerns and challenges, (2) the diagnostic assessment process, (3) receipt of the diagnosis, and (4) postassessment adaptations and needs. It was often reported that while caregivers were proactive in recognizing concerning behaviors and contacting health professionals, their concerns were frequently dismissed, and FASD was not considered or acknowledged by health professionals. Misconceptions about FASD and PAE by clinicians were reported in seven studies in Hayes et al. (2023) and stereotypes about facial features delayed FASD diagnosis. Despite barriers to accessing FASD assessment and feelings of grief and shame for some, families also report a sense of hope and relief, with benefits, including improved understanding of their child, as well as being a means to access appropriate and individualized support (Hayes et al., 2023).

By interviewing Australian psychologists, as well as the parents, caregivers, and adults who have lived experience of the assessment process, we aimed to better understand the current landscape of clinical practice assessing PAE and FASD. While lived experiences of FASD assessment have been studied (Hayes et al., 2023), the perspectives of psychologists conducting the assessments have been missed. By interviewing both groups, we aimed to integrate the perspectives and knowledge to understand current practices within Australia, in order to identify potential training gaps for psychologists.

## MATERIALS AND METHODS

### Research design

A semi‐structured interview guide was developed to examine contemporary lived experiences of FASD assessment, as well as the experiences of psychologists with FASD assessment. Semi‐structured interviews comprised three main areas: (a) experiences with FASD and PAE assessment, (b) training needs for psychologists, and (c) perspectives on universal screening of PAE.

Guiding questions are available in Appendix S1. These questions were guided by and expanded on the existing literature exploring attitudes of clinicians and those with lived and living experience of FASD assessment (Bagley & Badry, 2019; Bell et al., 2016; Corrigan et al., 2017, 2019; Crawford‐Williams et al., 2015a, 2015b; Kerimofski et al., 2024; Roozen et al., 2020). Questions and themes from these studies were reviewed, and a consensus decision was made about the interview questions. Additionally, a question was developed regarding universal screening of PAE following a recommendation for screening during infant health assessments and upon children entering child protection and justice systems from a coronial investigation in WA highlighting the link between FASD and suicide (Fogliani, 2019).

Participants were interviewed through video calls, which were recorded and transcribed, then analyzed with NVivo. The research was undertaken with ethics approval from the UWA Human Research Ethics Committee (2021/ET000667).

### Participants

The study had two groups of participants: (1) psychologist participants (PP) and (2) lived/living experience participants (LEP), including people with FASD and parents/caregivers who experienced the FASD assessment process. PP were recruited via professional networks, including past and present students of a graduate training program for FASD diagnosis in Australia, the Australia New Zealand FASD Clinical Network, LinkedIn, Facebook psychology groups, and the University research repository page of the co‐authors. LEP were recruited via the National Organisation for Fetal Alcohol Spectrum Disorders (NOFASD Australia) and were a mixture of biological parents, caregivers, and individuals with lived and living experience of FASD and FASD assessment. NOFASD Australia runs a parent, carer, and individuals with lived experience Expert Advisory Group (PEAG) with members asked whether they would like to participate in the project. These members were contacted, and additional people with lived experience were recruited via professional networks.

Participants were 10 psychologists and nine people with lived experience, biological parents, caregivers, and adults with FASD. One psychologist also had lived experience as a caregiver and was interviewed with both sets of questions. In the Lived Experience group, there were two adults with FASD, seven caregivers of a child or children with FASD, and one biological parent of a child or children with FASD. Two members with lived experience identified as Aboriginal, and no psychologists identified as Aboriginal or Torres Strait Islander.

### Data collection

Participants received an information sheet that prefaced the interview information to be collected prior to their interviews. Verbal consent was obtained prior to the interview and recording, in addition to completing a separate consent form and answering demographic questions via Qualtrics. Data collection from this study is part of a larger project, and demographics were collected to compare responses. Participants answered open‐ended questions, and interviews lasted approximately 1 h. Lived experience participants received a $50 e‐gift card to reimburse them for their time.

### Data analysis

Interview recordings were transcribed and de‐identified. NVivo was used to analyze interview responses and code for themes. Thematic analysis (Braun & Clarke, 2006) was employed, with six phases: (1) data familiarization, (2) generating initial codes, (3) searching for themes, (4) reviewing themes, (5) defining and naming themes, and (6) producing the report. Team members collaborated on coding, with two team members (KK & KP) independently coding two questions for all participants (approximately 30 per cent of the data corpus) and achieving an agreement goal of group consensus through intensive discussion (Harry et al., 2005).

## RESULTS

Results are presented across five key themes for PP and LEP: (1) stigma and stereotypes of PAE, (2) support for universal screening of PAE, (3) differential, co‐occurring, and missed diagnoses, (4) lack of support following diagnosis, and (5) need for improved training for psychologists.

### Stigma and stereotypes of PAE

It's actually much more prevalent than you might think. It does not just occur in Aboriginal people. And there are … the sort of like Champagne triangle of the upper‐class white women that think that it's OK to drink during pregnancy. (PP4)

The myths and stereotypes about drinking in pregnancy were discussed by both groups of participants. Participants were well informed that “higher educated, older, higher SES women” (PP7/LE1) were most likely to continue to drink in pregnancy. “People think that, you know, if there's no facial features, it's not FASD. People think it only affects low SES populations or only affects Aboriginal populations” (PP1) “A lot of them, believe it or not, are not Aboriginal women” (LE10).

#### Experiences with PAE assessment

Most psychologists reported that their current practice involves asking about PAE as part of a medical history during a developmental interview. Half of the psychologists interviewed reported using the AUDIT‐C (Bush et al., 1998) to quantify alcohol use, once they have established rapport. Normalizing alcohol use in context was a strategy used to understand what was happening in a person's life when they became pregnant. “We tend to drink a lot. So again, you know, I'm trying to sort of normalize the fact that this is Australian culture. There's nothing special about people drinking a lot when they're not intending to get pregnant … that could be any of us” (PP7/LE 1).

The experience of PAE assessment varied for biological parents compared with caregivers. One biological mother reported informing her GP about two or three binge drinking occasions prior to discovering the pregnancy and being advised to not “drink at that level again, now that you know you're pregnant” and then she “was never asked again. Obviously, it was never discussed and the number of clinicians throughout my son's first 13 years when I was asking questions, nobody ever asked me about alcohol, and I never thought to mention it” (LE5). For caregivers, some were aware of the biological mother's substance use (including alcohol) when their children were placed in their care or they reported being “lucky” (LE4) that PAE was documented in medical, police or child protection records. The frustrations of a lack of documentation or birth mothers denying alcohol use due to child protection concerns were also raised across both groups. Participants also reported concern that “There are still doctors who are recommending women have a drink. Calm your nerves, settle you down” (LE7).

#### Concerns and barriers to asking about PAE

A number of concerns and barriers were raised, including the need to be sensitive when asking about PAE and not wanting to shame the biological mother. Psychologists and caregivers also spoke of the difficulties in getting confirmation about PAE, either where there are doubts about the information provided by the birth mother or in cases where PAE is being confirmed through medical records or through other family reporting on the birth mother's drinking history. “When you ask that question, did you drink during pregnancy? … They'll often say no. And what they also mean is that the minute I found out that I was pregnant, I stopped drinking” (PP7/LE1). “There is a really deep problem with the birth mothers in admitting that they drink. So a lot of people either don't believe that it's a problem. It doesn't matter if I have a couple of glasses on the weekend, it's not gonna matter. They don't believe the one glass can do damage story” (LE7).

For the psychologists working in private practice, FASD is not a referral they report receiving. Clients will be referred with ADHD, learning, and behavioral queries, but the possible impact of PAE is not considered by referring families, pediatricians, or psychiatrists. One psychologist working in the private and public sector found that in “private practice, if I approach that question, it's kind of like, why are you asking? …Of course not. Why would I?” (PP9). “I think that they're very afraid, particularly with white upper‐class women, to even bring it up because these women react very poorly because they think that it is a lower socioeconomic status Aboriginal problem in society that doesn't concern them and that could not possibly happen to them or their child” (PP4). Some psychologists were also actively discouraged by supervisors during their university training to ask about PAE.

### Support for universal screening of PAE

I think it should 100% be compulsory. I think that GPs and obstetricians who do not ask this are bordering on breaching a duty of care to an unborn child and to a mother. (PP4)

**FIGURE 1:**
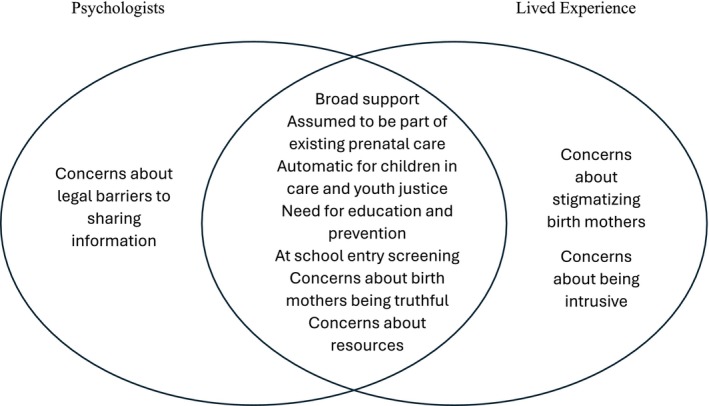
Thoughts about universal screening of prenatal alcohol exposure. The overlap in the center indicates shared ideas and concerns by both groups.

There was broad openness to universal screening of PAE among both psychologists and lived experience participants. See Figure 1 for an example of supports and barriers for universal screening of PAE among psychologists and lived experience participants. Some participants assumed that pregnant women are already routinely being asked about alcohol consumption during prenatal care and being given education about reducing PAE to minimize potential harm. Concerns were raised about the logistics of routine screening, with methods, such as blood tests and breathalyzers considered invasive, while concerns about women being truthful in reporting their alcohol consumption were also raised. The difficulties of recording and sharing this information were reported as mother and child records are kept separately, and public and private systems vary considerably. A protocol for obstetricians that included screening and education about alcohol, tobacco and substance use was discussed as ideal routine care. “I would hate to see like a potential opportunity to prevent FASD get missed because a doctor's uncomfortable asking a hard question” (PP1).

#### Clinician barriers

The clinician's own relationship with alcohol was discussed as a potential reason for not asking about PAE. “I think why there's so much resistance is because health professionals are, they're just people and they have their own relationship with alcohol. They probably know people who have drunk in pregnancy, their parents probably drank in their pregnancy. They might have had drinks while they were pregnant. And so they're not going to go. Oh my God, this is really harmful. This is what it does and tell other people. Because it actually affects their own, their personal feelings about the choices they make, and therefore so you actually have to deal with the health professionals, personal opinions and ideas about alcohol and that's really hard” (LE3). High risk exposure was discussed in the context of binge drinking in Australian culture “But when you think about high risk exposure, all you actually really need to get a 5 on the AUDIT is 1 binge drinking episode and binge drinking, you know it's less than a lot of us would think, I guess in Australia like I know that for me it's not unusual if I have like something on a Saturday night to have 4 glasses of wine” (PP1).

#### Screening in certain populations

Particular populations, such as children in care and youth involved in the justice system, were discussed as a priority for routine screening of PAE and FASD, with participants in both groups suggesting screening via the child health nurse in early years or as part of at‐entry school screening. The associated costs were discussed by both groups, along with the need for a focus on education and prevention, given the considerable costs of FASD.

### Differential, co‐occurring and missed diagnoses

It's been one heck of a journey. And it's still going. (LE10)

Both the psychologists and lived experience groups discussed differential, comorbid and missed diagnoses. For the lived experience groups, additional diagnoses included anxiety, autism, ADHD, intellectual disability, depression, dyslexia, dyscalculia, as well as experiences of gender dysphoria, psychosis and trauma. Some children were diagnosed early with global developmental delay, with caregivers reporting a reluctance on behalf of the pediatricians to diagnose FASD and preferring to diagnose GDD or Autism because “it's a lot easier to get that type of diagnosis, yeah, than it is to do the multidisciplinary approach to FASD” (LE10).

The lived experience group participants reported early signs that their child was not developing typically, including physical health concerns, as well as developmental delays and difficult behavior. Some caregivers began to question FASD early, based on known PAE, with some children in the group diagnosed prior to starting school. Other parents and caregivers reported “knowing there was something” (LE5) but being dismissed by clinicians. “I was made very much in those early years to feel like an anxious parent, a helicopter parent” (LE5). “CAMHS (Child and Adolescent Mental Health Services) just wiped it off as bad parenting” (LE7).

The sense of missed diagnoses was also reported by psychologists, who reported that “Once you've done the FASD training, you then look back on … kids that you've worked with or done assessments for and you kind of realize … oh yeah, I totally missed that. This is actually FASD happening for them” (PP3). The idea that the more diagnoses a child has, the more likely it is to be FASD was discussed frequently. “I think it would be good for psychologists to know, like, hey, if you have a child with this shopping list of diagnoses that includes ADHD, language disorder, oppositional defiant disorder, you know, depression, anxiety, … maybe it's a good idea to start thinking about FASD” (PP1). The continued stigma and varied presentation of FASD were discussed as a reason that psychologists feel other clinicians “doubt the validity of it (FASD) as a diagnosis” (PP1).

#### Barriers to assessment and diagnosis

Additional difficulties of getting diagnosed included a lack of FASD‐informed clinicians, difficulties confirming PAE, and the length of time it took from discovering PAE and beginning to query FASD to getting a diagnosis. “It took five years from that point to actually obtain the diagnosis” (LE3). For the adults with FASD, it was particularly difficult to find clinicians that would assess adults, as most Multidisciplinary Team (MDT) clinics in Australia are designed to support children. The adults had to self‐source assessments via several different clinicians, most of whom reported having no FASD knowledge or training. One adult was ultimately diagnosed by a specialist pediatrician. One adult reported being turned away by a university clinic for psychological assessment, being told “Your case seems complex, and I don't think we can help you” (LE2).

#### Diagnosing FASD and autism in aboriginal populations

Misdiagnosis of Autism and FASD was discussed by both groups, with psychologists discussing an article by a prominent Aboriginal psychologist that “FASD is damaging to Aboriginal communities” (PP1). The same participant reported that “my hunch is that FASD is probably just as common in white populations as it is in Aboriginal populations probably just diagnosed less, but I don't think the answer is like, stop diagnosing it in Aboriginal populations.” This difference in diagnostic formulation was also reported to be seen in Pediatricians—“I've heard a couple of pediatricians say quite openly. You know, there's a lot of non‐Indigenous children with autism diagnosis, and yet it's strange ‘cause the strategies don't quite fit. There seems to be so much more that's happening” (LE5).

### Lack of support following diagnosis

#### Mixed feelings following diagnosis

For some participants, there was a sense of relief following a FASD diagnosis, “Once I got the diagnosis and once, I understood that it was outside of my control to for like certain functioning was outside of my control and that I could get help to fix it or that … it was a weight off my shoulders. I was deeply depressed, and depression lifted straight away” (LE3). Others spoke of the isolation, shame, and a lack of understanding from the people around them, as well as clinicians involved in their care or the care of their children. There was a sense that individuals were receiving a diagnosis and then needing to find their own supports because “their job is about assessment” (LE4). It was widely agreed that a diagnosis did not guarantee support, with participants citing a lack of NDIS (National Disability Insurance Scheme) and school‐based funding. “A lot of kids don't get their plans (funding) for FASD” (LE6).

#### School supports and experiences

Parents and caregivers spoke about their need to advocate for their children, particularly in the school setting. “Unless the parent is prepared to get up and actually help educate people from within the school themselves, there's not really any stuff put into place for that to happen” (LE10). There was a widespread lack of understanding of FASD in schools, with parents and caregivers reporting they were the ones to educate the teachers and school leaders about FASD in order to support their children. Issues with engagement and supportive planning were also highlighted, with a sense that because FASD was not recognized as a funded disability across most school systems, their children were being disadvantaged. “The school system is not set up for them” (LE8).

School psychologists were discussed by both groups, with varied experiences. One caregiver described them as being “so overworked and they're not actually able to do assessments” (LE8). Another felt that “school psychologists actually can have a lot of influence in being able to get the right supports in place in schooling for these kids, because schooling is such a massive issue, it really is” (LE4). Psychologists had mixed feelings about school psychologists conducting assessments. “I had several conversations with school psych(ologist)s last year who told me that FASD did not exist, and they said it's not a thing. It's just ADHD or it's just autism, or it's just a really complex psychiatric case. They've probably got PTSD. And they absolutely do not think that FASD is a condition and those children are going to be severely disadvantaged if that's the psych(ologist) that is assessing them” (PP4). One participant working in a school does assess for FASD but was clear “it's not our role we've been told to hypothesize that it could be FASD. You would ask in the history taking, but it's not our role because the sensitive nature of it, that it must come from a medical forum first. So if I get a referral from pediatricians here, then I will go ahead with that. But it doesn't occur the other way around” (PP6).

#### Lack of support for secondary conditions associated with FASD

One caregiver highlighted the lack of support for both individuals and caregivers. “Lack of support for carers and parents to help us with how to manage it. Because it is such a difficult disability to manage. The life expectancy for these kids, for people with FASD is 35 because they commit suicide. These are very, very at risk people. Probably the most at risk people in the society” (LE7). One adult highlighted the difficulty of their employer becoming aware of their FASD diagnosis and the stigma and stress involved. Additional difficulties included children understanding and accepting their diagnoses, particularly as they became teenagers. Secondary impacts became more prevalent in this age group, including needing support to live independently, suicide attempts, significant mental health support needs, and difficulties with the law. For the psychologists who do assess FASD, they did not also offer therapy services. “The assessment is one part of it. But then it's kind of like, OK, well, where to from here? You know, what supports are available and how do I actually provide accommodations for this young person?” (PP5).

### Need for improved training for psychologists

I remember thinking when I was at the training, I remember thinking back to past assessments I've done and going, Oh my God, I can't believe I missed that, that's what was going on for that child. (PP3)

Both groups discussed the lack of training at university for psychologists and other health professionals and the importance of training more FASD‐informed professionals. The psychologists interviewed reported having at most one workshop during their Master's degree that included FASD, with no information about FASD or PAE discussed at an undergraduate level. Some psychologists were told explicitly by supervisors in their courses not to ask about PAE. “My adult and pediatric internal supervisors did not provide any training in that and one of them suggested that we don't even ask” (PP4). Additional barriers include not being in the DSM (APA, 2022) and debate about whose role it is to assess and diagnose FASD. Most psychologists reported their current training and knowledge of FASD being due to their own research, attending professional development or having a supervisor that was passionate about the impact of PAE. The psychologists wanted training to include information on appropriate supports and interventions, when to assess and when to refer on and when to consider FASD in their case formulation for both assessment and therapy clients.

For both groups, there was debate centered around single clinician versus MDT diagnosis. While some psychologists were trained to assess facial features, they expressed a preference for input from a pediatrician and other allied health clinicians. “Although I technically can do it, I don't like to do things like measure head circumference or measure facial (features). I don't feel that that's within my scope and I would prefer to leave that to someone who is an expert in doing … the medical and obviously refer for genetic testing if we need to rule out other genetic syndromes. They can diagnose any … comorbid neurological stuff… I think they need to have a role in it. I don't want to see it turn into a single clinician diagnosis the way autism has” (PP1). For those with lived experience, “a one stop shop would be ideal” (LE10), although both groups spoke directly of experiences where FASD had been overlooked by MDTs.

Additional discussion around training included differing opinions about whose role it is to assess and diagnose FASD. Some psychologists expressed that “neuropsych(ology) is specialized and people who don't have neuropsych(ology) training, I see them do (assessments) badly… But I think that to expect every single psych to do (assessment) is a bit of a big ask” (PP1). Other psychologists were more open to upskilling psychologists with general registration or other areas of endorsement. “I think, that there needs to be better training not only for neuropsychologists in FASD, but also anybody who is when it's within their scope to do an assessment, such as a school psychologist, because they're expected to by their school and understand what they're administering and how to interpret the findings and how to report on it, and also knowing when to refer on” (PP4). The LE participants were less focused on the endorsement of psychologists and more focused on clinicians being FASD‐informed. One adult reported that despite seeing “a well‐respected and prolific working neuropsychologist,” she “probably was aware of FASD, but she was not someone that could assess and make a diagnosis of FASD,” highlighting the need for further training across endorsements.

#### Training priorities from a lived experience perspective

The lived experience group had some key priorities for FASD training. “Rapport building is everything and that psychological safety and physical safety of that person coming into that interaction” (LE5). Understanding FASD through the lens of “brain‐based behavior” (LE10) and respecting lived experience were critical. “It's not just a foster child to me. I'm invested in making a difference for their futures. I don't have any specialized training, but please acknowledge me as someone that has his best interests at heart and work with me. And don't just dismiss me because you have that training and expertise” (LE9).

Additionally, they stressed the uniqueness of individuals with FASD and the stereotypes that persist around facial features. “Still on the NOFASD helpline, you'll have parents who've been waiting for six months, nine months, to get to see a pediatrician, and then they get told when they see the pediatrician, that it, well, it can't be FASD ‘cause they don't have facial features. That's still happening today” (LE5).

## DISCUSSION

This exploratory study aimed to understand the current landscape of PAE and FASD assessments in Australia for psychologists, as well as parents, caregivers, and individuals with FASD. Participants across both groups appeared to have a good understanding of the impact of PAE and the stereotypes about drinking in pregnancy, particularly the populations most at risk of FASD, as well as sharing concerns about the need for psychological safety and building rapport to try and reduce shame and stigma when asking about PAE.

### Universal screening of PAE

The Australian drinking culture was discussed as a way for psychologists to reduce shame and provide context for the normalization of alcohol consumption, particularly prior to discovering a pregnancy. In Australia, alcohol consumption is widespread, although alcohol consumption in pregnancy is declining, from 42% of women aged 14–49 in 2013 to 28% in 2022–2023, and for those who consumed alcohol in pregnancy, 64% did not know they were pregnant (NDSHS 2022–2023, 2024). Addressing and reducing PAE is an important issue in public health, given the social and economic costs associated with secondary conditions of FASD (Greenmyer et al., 2018; Popova et al., 2016). Only 47% of women were advised not to consume any alcohol, typically by a doctor, nurse, or midwife (NSDHS 2022–2023, 2024). This echoes the findings of Payne et al. (2005) that only 45% of health professionals routinely ask about alcohol use in pregnancy, as well as the results from the lived experience group who reported not being asked about PAE or being given misinformation that it is safe to drink certain amounts or at certain times in pregnancy. Clinicians, such as obstetricians, midwives, and GPs would likely benefit from training on the importance of screening for PAE, as well as how to ask about PAE in a sensitive and culturally informed way for all pregnant women, in order to increase the number of women asked about PAE and ultimately reduce PAE.

Participants from both groups recommended all women should be asked about PAE and the information recorded in medical records during routine antenatal care. The AUDIT‐C (Bush et al., 1998) is an appropriate tool to address and record PAE; however, participants noted that there would need to be careful consideration around how this information is then used. Opportunities were also identified to screen for PAE and FASD in early child health settings and at school entry. For example, in Victoria, maternal and child health nurses were trained to use the Social Attention and Communication Surveillance (SACS)‐Revised and SACS‐Preschool tools during well‐child checkups at 12, 18, 24, and 42 months of age for early autism detection, with the SACS‐R having an 83% positive predictive value and 99% negative predicted value of identifying autism (Barbaro et al., 2022). Screening tools for FASD exist; however, a systematic review by Grubb et al. (2021) identified most have weak psychometric properties and have a significant risk of bias. Thus, there is a need for further research to develop clinically validated screening tools.

### Limited diagnostic capacity

Both groups identified limited diagnostic and support services in Australia, consistent with Hayes et al. (2023) systematic review of FASD assessment experiences. The lived experience group highlighted concerns around the lack of FASD‐informed clinicians leading to missed diagnoses, as well as long journeys to assessment. In Australia, the demand for FASD diagnosis is far greater than the availability of diagnostic services, with no consistency in the model of service (Panton et al., 2022). While the traditional multidisciplinary team (MDT) approach is considered the gold standard (Bower et al., 2016), training clinicians outside of this approach can improve access to assessment and diagnosis (Burd & Popova, 2019). In particular, the adults with FASD interviewed highlighted that there were no MDTs for adult FASD assessment in Australia, and not all states have dedicated FASD clinics.

### Developing FASD training

In addition to a lack of diagnostic services, participants with lived experience highlighted the lack of FASD‐informed clinicians available to offer therapeutic support. The psychologists interviewed for this study considered FASD to be a niche area of both assessment and intervention, with the psychologists' knowledge about FASD coming from professional development that they had sought out or from an informed supervisor at university. Both groups stated the importance of embedding teaching about the impact of PAE on neurodevelopment at both undergraduate and postgraduate levels in Australia. For practicing psychologists, there have been recommendations to allocate government funding for training in FASD assessment, test interpretation, and diagnosis (Commonwealth of Australia, 2021; Reid, 2018; Reid et al., 2020). Online training has previously been reported to be the preferred option for Australian psychologists; however, this was in the context of psychologists who already work in the field (Kerimofski et al., 2024).

When considering developing further FASD training, there was an emphasis from participants on recognizing that assessments and interventions should be individualized. Culture was not discussed directly by participants in the interviews; however, the FASD Indigenous Framework (Hewlett et al., 2023) suggests ways for non‐Aboriginal clinicians and Aboriginal peoples to access healing‐informed, strengths‐based, and culturally responsive FASD assessments and supports. The framework establishes elements of knowledge for clinicians, “knowing,” such as understanding the legacies of colonization and Aboriginal perspectives, as well as ways of being, “unlearning” and doing, “yarning” and applying a strengths‐based approach and advocacy.

Additional consideration for future training should be given to the diagnostic terminology. Eliason et al. (2024) raised concerns that the term FASD contributes to a culture of racism and discrimination and should be revised to a broader diagnostic term, such as “neurodevelopmental disorder” as it is difficult to confirm that PAE is the primary cause of neurodevelopmental impairment. Others argue that by replacing the term FASD, the FASD community may be further marginalized and the important public health messaging about the risks of PAE will be impacted (Kachor, 2024). Currently, there is no unified diagnostic approach, with more than 10 different diagnostic criteria globally (Reid et al., 2022). Inclusion in the next edition of the DSM (APA, 2022) could support a cohesive approach to diagnostic criteria and terminology.

## LIMITATIONS

This study was limited by the sample size and generalizability of results. The lived experience participants were derived from the NOFASD PEAG and are actively involved in support and advocacy for FASD. They are not necessarily representative of all individuals, parents, and caregivers affected by FASD.

Similarly, the psychologists interviewed had a higher baseline level of FASD knowledge than psychologists previously surveyed in Kerimofski et al. (2024). This was helpful in terms of achieving productive discourse about FASD and their experiences but may not reflect most Australian psychologists' experiences.

## CONCLUSION

The importance of asking about PAE in a sensitive manner, including universal screening opportunities, was highlighted by both groups of participants. A call to increase the number of FASD‐informed clinicians to offer both assessment and individualized support will require embedding knowledge about FASD and PAE within all university training programs for psychologists, as FASD is currently considered a niche area of assessment, with clinicians holding outdated views at times. Future research could focus on developing culturally appropriate and sensitive screening tools for PAE and FASD, as well as training for psychologists in these assessments to improve early identification and support. Additionally, the broad support for universal screening of PAE suggests training clinicians involved in antenatal care to consistently screen for PAE in a sensitive and culturally informed manner.

## FUNDING INFORMATION

This research received no specific grant from any agency in the public, commercial or not‐for‐profit sectors.

## CONFLICT OF INTEREST STATEMENT

The authors declare no conflict of interest.

## Supporting information


Appendix S1

## Data Availability

Data will be made available upon request.
