# Supplementary material for: Fetal alcohol spectrum disorder identification in Australia: A qualitative analysis of perspectives from psychologists and individuals with lived and living experience
Source: Alcohol Clin Exp Res (Hoboken). 2025 Mar 31;49(5):1042–52. doi: 10.1111/acer.70040 (PMC12098810; doi:10.1111/acer.70040)
Supplement: Supplementary file 1 — Appendix S1 [file ACER-49-1042-s001.zip › acer70040-sup-0001-Guiding Questions and Themes Psychologist Participants.docx]

| Question/Area of Exploration | Source |
| --- | --- |
| **What is your experience with FASD assessment?**  -working with individuals and/or families?  -assessment?  -personal experience? | Expansion of survey |
| **What is your understanding of the impact of consuming alcohol in pregnancy?**  -personal experience  -professional experience  -in society  -source of knowledge | Expansion of survey; (Bagley & Badry, 2019; Crawford-Williams et al., 2015a, 2015b) |
| **What is your experience assessing for PAE?**  -when do you assess?  -how do you assess?  -what holds you back from asking about PAE?  -stigma most common concern- your understanding/view  -if they assess, what practices do you use to mitigate harm to biological mother when asking? | Expansion of survey; (Crawford-Williams et al., 2015a) |
| **What are your training needs in order to A. more consistently and confidently assess for PAE in clinical interviews**, **B improve confidence in the selection and interpretation of psychometric assessments?**  -current training completed?  -if psychologist does not engage in assessments, would they be interested in training around FASD to support clients in therapy? | Expansion of survey |
| **What are your thoughts on universal screening of PAE?**  -in what setting  -high risk groups  -at what age | (Fogliani, 2019) |

Questions in bold will be asked of participants with prompts underneath that may be used to ensure the necessary information is captured by the interviewee.

Bagley, K., & Badry, D. (2019). How Personal Perspectives Shape Health Professionals' Perceptions of Fetal Alcohol Spectrum Disorder and Risk. *Int J Environ Res Public Health*, *16*(11). <https://doi.org/10.3390/ijerph16111936>

Crawford-Williams, F., Steen, M., Esterman, A., Fielder, A., & Mikocka-Walus, A. (2015a). "If you can have one glass of wine now and then, why are you denying that to a woman with no evidence": Knowledge and practices of health professionals concerning alcohol consumption during pregnancy. *Women Birth*, *28*(4), 329-335. <https://doi.org/10.1016/j.wombi.2015.04.003>

Crawford-Williams, F., Steen, M., Esterman, A., Fielder, A., & Mikocka-Walus, A. (2015b). "My midwife said that having a glass of red wine was actually better for the baby": a focus group study of women and their partner's knowledge and experiences relating to alcohol consumption in pregnancy. *BMC Pregnancy Childbirth*, *15*, 79. <https://doi.org/10.1186/s12884-015-0506-3>

Fogliani, R. (2019). *Inquest into the deaths of thirteen children and young persons in the Kimberley region, Western Australia*. Coroner's Court of Western Australia.
