# Supplementary material for: Fetal alcohol spectrum disorder identification in Australia: A qualitative analysis of perspectives from psychologists and individuals with lived and living experience
Source: Alcohol Clin Exp Res (Hoboken). 2025 Mar 31;49(5):1042–52. doi: 10.1111/acer.70040 (PMC12098810; doi:10.1111/acer.70040)
Supplement: Supplementary file 1 — Appendix S1 [file ACER-49-1042-s001.zip › acer70040-sup-0002-Guiding Questions Lived Experience Group.docx]

| Question/Area of Exploration | Source |
| --- | --- |
| **What is your experience with FASD assessment?**  -positive experiences  -negative experiences  -experience of stigma (different kinds of stigma) | Expansion of survey; (Bell et al., 2016; Corrigan et al., 2017; Corrigan et al., 2019; Roozen et al., 2020) |
| **What is your experience with PAE assessment?**  -how did the psychologist/professional assess PAE?  -what (if anything) did the psychologist/professional do to promote psychological safety when asking about PAE?  -positive experiences  -negative experiences  -experience of stigma (different kinds of stigma) | Expansion of survey; (Bell et al., 2016; Corrigan et al., 2017; Corrigan et al., 2019; Roozen et al., 2020) |
| **What (if anything) would you change about the FASD assessment process?** | Expansion of survey |
| **What (if anything) would you change about the PAE assessment process?**  -how could the psychologist promote your safety and interests when asking about PAE? | Expansion of survey |
| **What would you like psychologists to know about working with people with FASD?** | (Bell et al., 2016; Corrigan et al., 2017; Corrigan et al., 2019; Roozen et al., 2020) |
| **What are your thoughts on universal screening of PAE?**  -in what setting  -high risk groups  -at what age | (Fogliani, 2019) |

Questions in bold will be asked of participants with prompts underneath that may be used to ensure the necessary information is captured by the interviewee.

Bell, E., Andrew, G., Di Pietro, N., Chudley, A. E., N. Reynolds, J., & Racine, E. (2016). It’sa shame! Stigma against fetal alcohol spectrum disorder: Examining the ethical implications for public health practices and policies. *Public Health Ethics*, *9*(1), 65-77.

Corrigan, P. W., Lara, J. L., Shah, B. B., Mitchell, K. T., Simmes, D., & Jones, K. L. (2017). The public stigma of birth mothers of children with fetal alcohol spectrum disorders. *Alcoholism: Clinical and Experimental Research*, *41*(6), 1166-1173.

Corrigan, P. W., Shah, B. B., Lara, J. L., Mitchell, K. T., Combs-Way, P., Simmes, D., & Jones, K. L. (2019). Stakeholder perspectives on the stigma of fetal alcohol spectrum disorder. *Addiction Research & Theory*, *27*(2), 170-177.

Fogliani, R. (2019). *Inquest into the deaths of thirteen children and young persons in the Kimberley region, Western Australia*. Coroner's Court of Western Australia.

Roozen, S., Stutterheim, S. E., Bos, A. E., Kok, G., & Curfs, L. M. (2020). Understanding the social stigma of fetal alcohol spectrum disorders: From theory to interventions. *Foundations of Science*, 1-19.
